# High throughput sequencing analysis of RNA libraries reveals the influences of initial library and PCR methods on SELEX efficiency

**DOI:** 10.1038/srep33697

**Published:** 2016-09-22

**Authors:** Mayumi Takahashi, Xiwei Wu, Michelle Ho, Pritsana Chomchan, John J. Rossi, John C. Burnett, Jiehua Zhou

**Affiliations:** 1 Department of Molecular and Cellular Biology, Beckman Research Institute of City of Hope, Duarte, CA, 91010, USA; 2 Integrative Genomics Core, Beckman Research Institute of City of Hope, Duarte, CA, 91010, USA; 3 Irell and Manella Graduate School of Biological Sciences, Beckman Research Institute of City of Hope, Duarte, CA, 91010, USA

## Abstract

The systemic evolution of ligands by exponential enrichment (SELEX) technique is a powerful and effective aptamer-selection procedure. However, modifications to the process can dramatically improve selection efficiency and aptamer performance. For example, droplet digital PCR (ddPCR) has been recently incorporated into SELEX selection protocols to putatively reduce the propagation of byproducts and avoid selection bias that result from differences in PCR efficiency of sequences within the random library. However, a detailed, parallel comparison of the efficacy of conventional solution PCR *versus* the ddPCR modification in the RNA aptamer-selection process is needed to understand effects on overall SELEX performance. In the present study, we took advantage of powerful high throughput sequencing technology and bioinformatics analysis coupled with SELEX (HT-SELEX) to thoroughly investigate the effects of initial library and PCR methods in the RNA aptamer identification. Our analysis revealed that distinct “biased sequences” and nucleotide composition existed in the initial, unselected libraries purchased from two different manufacturers and that the fate of the “biased sequences” was target-dependent during selection. Our comparison of solution PCR- and ddPCR-driven HT-SELEX demonstrated that PCR method affected not only the nucleotide composition of the enriched sequences, but also the overall SELEX efficiency and aptamer efficacy.

SELEX (systemic evolution of ligands by exponential enrichment) technology is a dedicated selection process for generating specific aptamers. Since the first SELEX experiments were described by three independent groups in 1990^1^,^2^,^3^, numerous nucleic acid aptamers have been raised to a wide range of targets^4^,^5^,^6^, including small molecules, toxins, peptides, proteins, viruses, bacteria, whole cells, and even live animals. These aptamers, often termed “chemical antibodies,” possess many comparable properties and even superior advantages over protein antibodies. Nucleic acid aptamers have been extensively explored for various applications in diagnostics^7^,^8^,^9^, biosensors^10^,^11^, imaging^12^,^13^, biomarker discovery^14^,^15^, therapeutics^16^,^17^, and targeted drug delivery^14^,^18^,^19^,^20^,^21^.

In parallel with application development, intensive efforts have been made to improve and optimize the aptamer-selection technique, in order to more efficiently identify the best possible aptamers. There are several crucial factors that contribute to overall SELEX performance, including a highly diverse initial single-stranded (ss) DNA or RNA library without an inherent bias, and effective partitioning, recovery, and re-amplification of bound sequences. Specialized technologies have recently been incorporated into the original SELEX process to modernize and improve overall selection efficiency. For example, high throughput sequencing (HTS) technologies and bioinformatics analysis combined with SELEX (HT-SELEX) facilitates the rapid identification of aptamers and provides a comprehensive landscape for the molecular evolution^22^,^23^,^24^,^25^,^26^,^27^. In the traditional SELEX approach, individual candidate aptamers are identified by cloning only the final, enriched aptamer library, followed by classic Sanger sequencing approaches. The benefit of HT-SELEX is that millions of sequence reads can be analyzed from each selection round and thus provide insight into the whole SELEX process. This comprehensive information, including primary sequences, total reads, frequencies of each unique sequence, distribution of each nucleotide in the random sequence, nucleotide composition, and rate of molecular enrichment, can guide the improvement of SELEX by enabling a better understanding of the SELEX progression and factors that impact selection^23^. Moreover, because the changes of aptamer family and round-to-round enrichment can be tracked throughout selection cycles, HT-SELEX is capable of identifying candidate aptamers with high affinity at a much earlier selection round, which can reduce the cost and time associated with over-selection, and avoid potential PCR artifacts in the re-amplification step^28^.

Most recently, droplet digital PCR (ddPCR) or emulsion PCR (ePCR) has been incorporated into the SELEX protocol to reduce the propagation of byproducts, which putatively avoids PCR bias and selection failure^28^,^29^,^30^,^31^. These systems are methods that compartmentalize and miniaturize the PCR reaction by generating a water-in-oil emulsion containing numerous droplets, which creates a local homogeneous amplification micro-environment. Such methods have shown capacity for maintaining library diversity and preventing the loss of potential aptamers that are difficult to amplify^30^,^31^. Yufa *et al*. have used ePCR in combination with nonequilibrium capillary electrophoresis of equilibrium mixtures (NECEEM), a homogeneous partitioning technique for DNA aptamer selection, to successfully isolate DNA aptamers; in contrast, no detectable enrichment was observed with conventional solution PCR in their model^31^. Unlike ePCR, where size and number of droplets cannot be controlled, resulting in a heterogeneous droplet emulsion, ddPCR utilizes a droplet generator to create an emulsion of uniformly sized droplets, so that DNA templates can be distributed equally in each droplet in a reaction^30^. Recently, Ouellet *et al*. reported a high-fidelity ddPCR-based SELEX strategy (Hi-Fi SELEX) for isolating high-affinity DNA aptamers, in which ddPCR was used to achieve high-fidelity amplification of DNA libraries in a bias-free manner^30^. They used indirect methods (quantitative PCR or fluorometric quantitation) to estimate the sequence diversity of retained pools, which suggested a preservation of the diversity and complexity in DNA libraries by ddPCR. However, a detailed, parallel comparison (e.g., of primary sequences, frequency of aptamer family, total reads, and binding affinity) of the impacts of solution PCR and ddPCR on the RNA aptamer-selection process is needed. Although these studies showed the advantages of ddPCR for aptamer selection, no direct sequence analysis of random DNA libraries generated by ddPCR has been reported.

To provide the structural diversity needed to identify specific aptamers, an initial, unselected RNA library should ideally have maximal sequence heterogeneity or randomness in its variable region. Individual sequences and individual nucleotides should be equally distributed in a library; a sequence bias in the initial library may misrepresent the sequence landscape, with the potential to adversely affect the overall selection. Although the nucleotide composition in initial RNA libraries has already been studied by other groups^32^,^33^,^34^, existence of sequence bias has never been discussed.

In the present study, we therefore used this powerful HT-SELEX technique to thoroughly investigate the influences of initial RNA library and PCR methods on RNA aptamer selection. Our analysis revealed that distinct “biased sequences” and nucleotide composition existed in the initial, unselected libraries purchased from two different manufacturers. The bias may have originated from solid-phase chemical synthesis. Interestingly, the “biased sequences” within the original library exhibited distinct fates during the selection, suggesting a target-dependent manner. Furthermore, in the solution PCR-driven SELEX, dramatic enrichment occurred as early as round 5, whereas ddPCR-driven SELEX showed slower molecular evolution with higher sequence diversity throughout the selection process. We observed a notable increase in thymidine in the solution PCR-driven libraries, compared to an enrichment of guanine-rich sequences in the ddPCR-driven selection. Taken together, our data suggest that PCR method affects not only the nucleotide composition of the initial RNA pools, but also the overall SELEX and aptamer selection efficacy. We demonstrated that HT-SELEX is a powerful and precise tool for guiding library design and aptamer selection.

## Results

### Bias in the sequence and nucleotide distribution is observed in the initial, unselected RNA library

**Figure 1:**
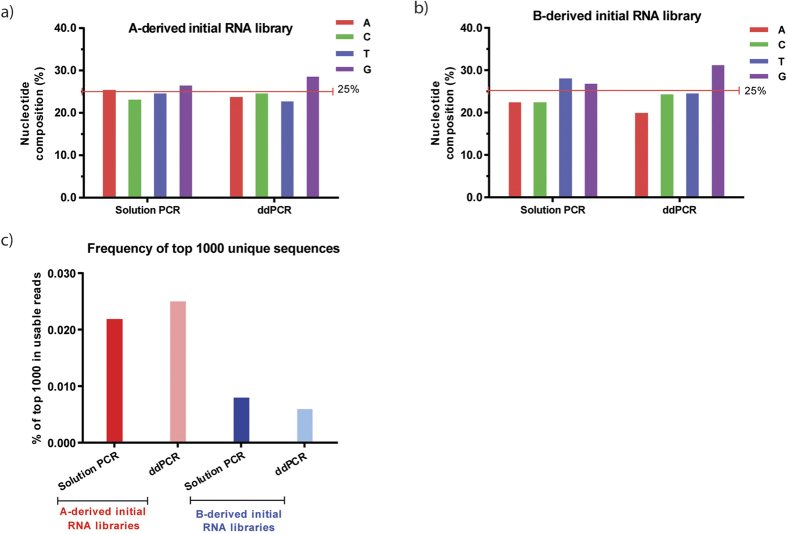
Nucleotide bias is observed in the initial, unselected RNA libraries. (**a**,**b**) An increase in guanines occurred in A-derived initial RNA libraries (**a**) as well as B-derived initial RNA libraries (**b**). (**c**) The frequencies of the most frequent 1,000 unique sequences in all the usable reads were identified.

To understand the impacts of initial ssDNA library source on aptamer library generation, we purchased two ssDNA template libraries from two different oligonucleotide manufactures (company A and B for clarifying). Each contained the same two constant sequence regions flanking a variable region of 30 nt. Per our request, the manufacturers chemically synthesized the libraries with the same proportion of each nucleoside (1:1:1:1) by hand mixing and desalted purification. To understand the effects of PCR methods on aptamer library generation, we generated double-stranded (ds) DNA libraries from the ssDNA templates by either conventional solution PCR or ddPCR amplification. We then converted the dsDNA libraries into the initial RNA libraries using *in vitro* transcription. Following HTS analysis, approximately 30–50 million useful total reads were obtained from each library. Although the overall nucleotide distribution in the sequenced RNA libraries did not significantly deviate from randomness (Supplementary Fig. S1), composition analysis in the 30-nt random region revealed nucleotide bias toward guanine-rich sequences (up to 6.2% when using 25% as average baseline) (Table 1 and Fig. 1a,b). The ssDNA library source did not appear to impact nucleotide distribution, as we observed an increase in guanines in both the company A-derived and company B-derived libraries, although to a varying degree (Table 1 and Fig. 1a,b). In contrast, PCR method did appear to impact nucleotide distribution, as ddPCR-converted RNA libraries contained relatively higher guanine content, while solution PCR-converted RNA libraries contained relatively higher thymidine content. These nucleotide biases may affect subsequent aptamer selection.

Next, we extracted the top 1000 unique sequences from the generated database for further analysis; useful reads and frequencies were tabulated (Table 2 and Fig. 1c). Surprisingly, although the percentages of usable reads were similar, the top 1000 unique sequences in the A-derived initial RNA libraries showed a 3-fold higher frequency (0.022–0.025%) than those in the B-derived initial RNA libraries (0.006–0.008%), regardless of the PCR method used. This suggests that the A-derived initial RNA libraries lack sequence complexity, while the B-derived initial RNA libraries have a higher heterogeneity. Thus, the B-derived initial RNA library contained fewer biased sequences than A-derived library – a feature that is likely to enhance the diversity and quality of aptamers obtained from subsequent rounds of SELEX.

Additional clustering analysis of the top 50 unique sequences revealed some predominant sequences in these RNA libraries. The reads and frequencies of the representative sequences in each cluster were tabulated (Tables 3 and 4). We observed similar frequencies between solution PCR- and ddPCR-converted RNA libraries, suggesting that PCR methods under our experimental conditions did not significantly change library heterogeneity. However, different predominant sequences were found in A- or B-derived RNA libraries, implying that chemical synthesis is important for these differences. A-derived RNA libraries contained four predominant consensus sequence groups in the top 50 unique sequences (Table 3). The most abundant sequence (Group 1) occupied 0.0117–0.0142% of total reads (or over 87% of the top 1000 unique sequences). In contrast, only one sequence group predominated in the B-derived RNA libraries, occupying 0.0003–0.0005% of total reads (or less than 5% of the top 1000 unique sequences) (Table 4). These observations confirm that A-derived RNA libraries have less sequence complexity, while B-derived RNA libraries contains more diverse sequences, which is preferable for aptamer selection. Taken together, our data suggest the PCR method chosen for conversion of the initial RNA libraries generates nucleotide bias, while the manufacturing procedure (solid-phase chemical synthesis) generates sequence bias.

### Aptamer selection and the fate of biased sequences is target dependent

To determine whether sequence bias in the initial, unselected RNA libraries adversely affects aptamer selection, we performed independent live cell-based SELEX experiments for two different targets, human CCR7 and CD2. Cell surface expression of CCR7 and CD2 on HeLa-CCR7+ and HeLa-CD2+ cells, respectively, was verified and monitored during the selections using flow cytometric analysis. Counter selection was conducted using HeLa cells that do not express the target protein(s). Detailed selection conditions are described in Materials and Methods.

**Figure 2:**
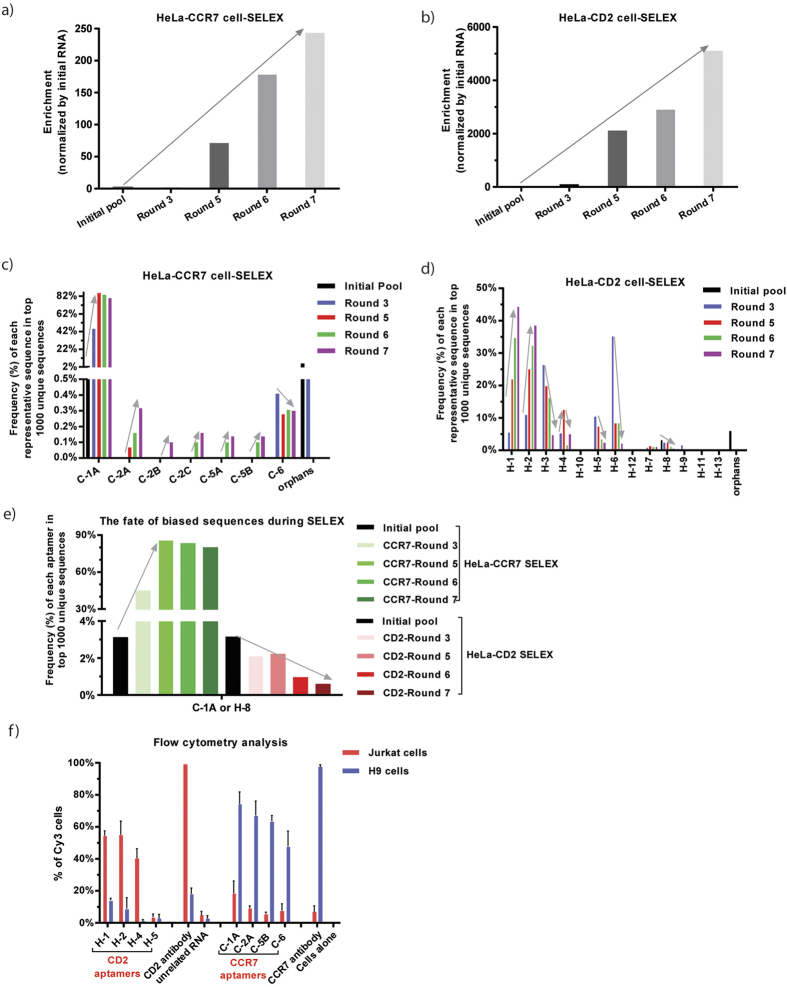
Evolution of CCR7 or CD2 RNA aptamers via live cell-based HT-SELEX. (**a**,**b**) The molecular enrichment at each round of HeLa-CCR7 cell-SELEX (**a**) and HeLa-CD2 cell-SELEX (**b**). The molecular enrichment at each round was calculated by the formula: total reads of top 1000 sequences in round X/unselected library (initial library). (**c**,**d**) The frequency of each group at each selection round of HeLa-CCR7 cell-SELEX (**c**) and HeLa-CD2 cell-SELEX (**d**). After alignment of the top 50 sequences, several groups of aptamers were identified. The percent frequency of each group at each selection round was calculated by the formula: the reads of each group/the total reads of top 1000 unique sequences. (**e**) The fate of biased sequence from initial RNA libraries during SELEXs. (**f**) Cell-type specific binding of selected RNA aptamers. Cy3-labeled RNAs were tested for binding to H9 (CCR7^high^CD2^low^) and Jurkat (CCR7^low^CD2^high^). PE-CF594-conjugated anti-human CCR7 antibody or PE-conjugated anti-human CD2 antibody was used to stain cellular surface CCR7 or CD2, respectively. Data represent the average of triplicate measurements. Error bars represent SD.

Using the B-derived initial RNA library, which shows higher sequence complexity as shown above, a total of seven selection rounds containing both positive and counter selections were performed for each cell-based SELEX experiment using the solution PCR protocol. To follow the SELEX progression, we focused on RNA pools recovered from rounds 3, 5, 6 and 7. These pools were subjected to HTS and bioinformatics analysis. The reads and frequencies of the top 1000 unique sequences were tabulated (Table 5) and used to calculate enrichment (Fig. 2a,b). The distribution of each nucleotide (A, T, C and G) at the 30-nt random region within the RNA sequence after each round is shown in Supplementary Fig. S2a,b. A dramatic increase in enrichment occurred after the fifth round of selection in each experiment (Table 5, Fig. 2a,b). We also observed that the molecular diversity was dramatically converged after round five, which reflects an increase in total reads of the top 1000 unique sequences, suggesting that some specific aptamer sequences were successfully enriched during the selection. The HeLa-CCR7 and HeLa-CD2 cell-SELEX experiments each showed robust evolution dynamics, but to a varying degree (243-fold enrichment versus 5106-fold enrichment after the seventh round of selection, respectively), indicating that the SELEX target plays a key role in the enrichment rate.

To identify enriched aptamer candidates, individual sequences were classified into groups based on alignment of the top 50 unique aptamer sequences and similarity of theoretical secondary structures predicted using RNA folding algorithm Mfold. Representative sequences from each group were selected for further analysis due to relative abundance within each group (Tables 6 and 7). Different aptamers were identified in the two SELEX experiments. We calculated the relative frequency to determine the evolution of each sequence group from the initial pool through rounds 3, 5, 6 and 7. In the HeLa-CCR7 cell-SELEX (Fig. 2c), most groups (e.g., C-1A and C-2A) showed a progressive increase from round 5 to round 7. Similarly, the HeLa-CD2 cell-SELEX (Fig. 2d) identified several highly enriched sequences with a progressive increase (e.g., H-1 and H-2). However, some sequences (e.g., H-3, H-5, H-6, and H-8) decreased and were even eliminated during selection. These results suggest that some nonspecific or lower-affinity species can be removed by selection pressure.

We next tracked the “biased sequence” which predominated the initial RNA library, and observed a distinct fate (Fig. 2e). In the HeLa-CCR7 cell-SELEX, the biased sequence named as C-1A in the CCR7 aptamer group showed the highest frequency and dramatic enrichment (from 3.16% to 80.37%). In contrast, the *same* biased sequence named as H-8 in the CD2 aptamer group was dramatically reduced during HeLa-CD2 cell-SELEX selection (from 3.16% to 0.63%). Given that the same selection protocol and the same initial RNA library were applied to both cell-SELEX experiments, we can conclude that the fate of the biased sequence depends largely on the SELEX target.

To further validate the effects of SELEX target on selection, several representative aptamers from each group were synthesized to evaluate their binding affinity for H9 cells (CCR7^high^CD2^low^) and Jurkat cells (CCR7^low^CD2^high^). Flow cytometric analysis revealed that the selected aptamers against CCR7 or CD2 specifically bound to the respective target cells (Fig. 2f). For example, aptamer C-1A, which showed progressive enrichment in HeLa-CCR7 cell-SELEX, selectively bound to CCR7^high^ H9 cells, suggesting it is selected specifically against CCR7. Taken together, these data show that the cell-SELEX target influences aptamer selection and that sequence bias in the initial RNA library may be overcome by the selection stringency.

### PCR methods affect sequence diversity and molecular enrichment during SELEX

As shown above, the PCR method chosen for conversion of the dsDNA library can affect nucleotide composition of the RNA library. To better understand the effect of PCR methods on the overall SELEX selection, we performed two parallel cell-based selections for human CCR7 target protein using solution PCR- and ddPCR-driven HT-SELEX. Except for PCR methods, the same selection protocol and materials were applied in the selections. The initial B ssDNA library or recovered cDNA libraries from each selection cycle were amplified by either solution PCR or ddPCR during the selections. We conducted seven selection cycles for both SELEX experiments and used HTS and bioinformatics analysis to examine the primary sequences of individual aptamers, their total reads, frequency of each cluster, and nucleotide composition and distribution of the RNA pools derived from several selection rounds (initial/unselected, 1, 3, 5, 6, and 7).

**Figure 3:**
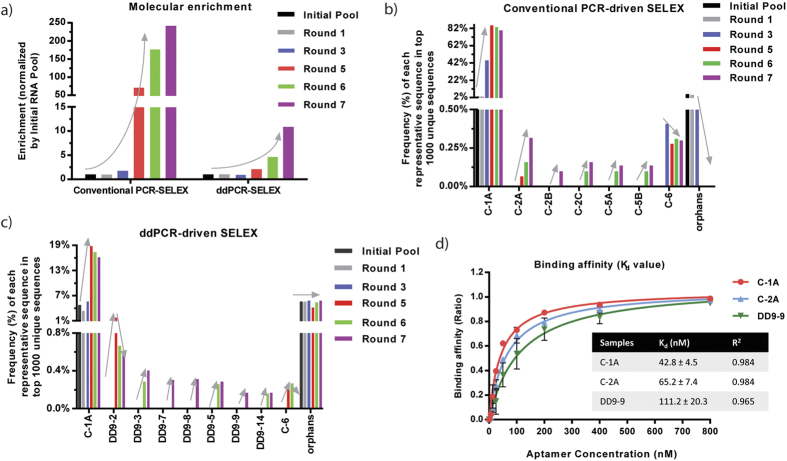
Evolution of CCR7 RNA aptamers via live cell-based HT-SELEX. (**a**) The molecular enrichment at each round of the solution PCR- and ddPCR-driven HT-SELEX. The molecular enrichment was calculated as described above. (**b**,**c**) The frequency of each group at each selection round of the solution PCR-driven SELEX (**b**) and ddPCR-driven SELEX (**c**). After alignment of the top 50 sequences, several groups of aptamers were identified. The percent frequency of each group at each selection round was calculated by the formula: the reads of each group/the total reads of top 1000 unique sequences. (**d**) Cell surface binding constant (K_d_) of selected CCR7 aptamers in H9 (CCR7 positive) cells was evaluated by flow cytometry. Data represent the average of triplicate measurements. Error bars represent SD.

The initial, unselected RNA libraries and round 1- and 3-selected RNA libraries showed similar total reads and frequencies of the top 1000 unique sequences (Table 8), suggesting that PCR methods did not contribute significant deviation in sequence diversity at the beginning or early round of the selection. In later rounds, we observed lower frequencies of the top 1000 unique sequences in ddPCR-driven SELEX compared to solution PCR-driven SELEX, suggesting that the ddPCR method preserves higher sequence diversity throughout the selection process (Table 8). Specifically, in the solution PCR-driven SELEX, dramatic molecular evolution was apparent as early as round 5, while ddPCR-driven SELEX showed slower molecular evolution (Fig. 3a). Despite the difference in evolution rate, we observed a sequence diversity convergence in additional selection rounds (e.g., round 5 to 7) in both SELEX experiments (Supplementary Fig. S3a).

To identify potential aptamers, we extracted the top 50 unique sequences for clustering analysis. Individual sequences were classified into several groups based on their primary sequences and predicted secondary structures. We identified four groups in the solution PCR-driven SELEX and seven groups in the ddPCR-driven SELEX, and tabulated reads and frequencies for representative sequences from each group (Tables 9 and 10). In both experiments, the sequence C-1A that predominated the initial RNA libraries was the most abundant, demonstrating the highest frequencies among all the aptamer candidates. Although two overlapped sequences (C-1A and C-6) were selected from both SELEX experiments, different aptamers were identified. Most of the selected aptamers showed a progressive enrichment beginning in round 5, while some sequences (e.g., DD9-2, C-6) were reduced and even went extinct, suggesting they may be lower-affinity binders (Fig. 3b,c).

To evaluate the binding affinity of these selected aptamers for the target protein CCR7, several representative sequences from each group were synthesized and used for flow cytometry and gel shift assays (Supplementary Fig. S3b). Flow cytometry analysis showed that the selected RNA aptamers were capable of binding selectively to the target-expressing cells (HeLa-CCR7, H9, and human memory CD4+ T cells), demonstrating that the selected aptamers are specific to the CCR7 target protein. Except for the overlapped candidate aptamer (C-1A), the top aptamer candidate raised from solution PCR-driven SELEX (C-2A) showed higher binding affinity (65.2 nM of K_d_) to CCR7-expressing cells than the top aptamer candidate from ddPCR-driven SELEX (DD9-9, 111.2 nM of K_d_) (Fig. 3d). Taken together, our data suggest that although ddPCR contributes to a greater sequence diversity of the initial RNA library, this diversity may compromise the evolution rate of aptamers, and an abundance of candidates may lead to difficulties in characterization^28^.

### PCR methods account for the observed nucleotide bias during SELEX

**Figure 4:**
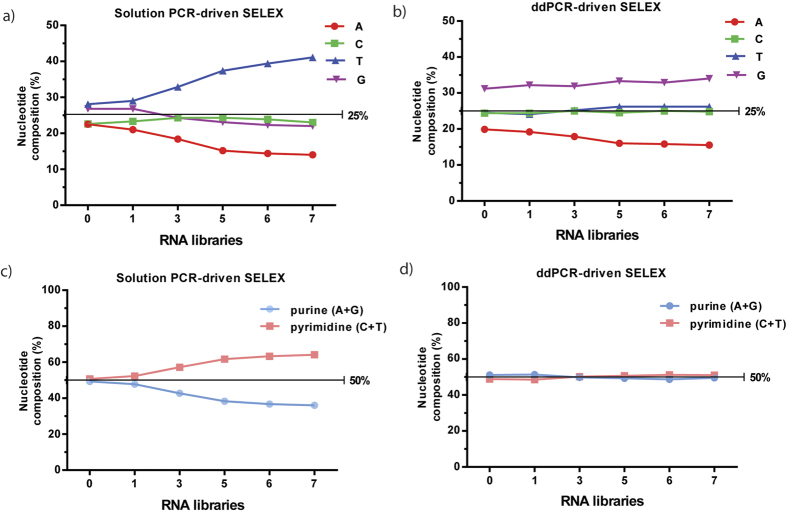
Nucleotide bias observed in the targeted selection. The nucleotide compositions (A, C, T, and G) in the random region showed different changes in solution PCR-driven SELEX (**a**) versus ddPCR-driven SELEX (**b**). A bias for pyrimidines over purines is observed in solution PCR-driven SELEX (**c**). The compositions of purine and pyrimidine were almost equal (around 50%) in the ddPCR-driven SELEX (**d**).

We have demonstrated that PCR methods cause nucleotide bias in the initial RNA libraries. Moreover, it has been reported that a bias toward pyrimidine (C/T)-rich sequences exists during SELEX^32^. To further investigate nucleotide bias, we analyzed the nucleotide composition of the RNA pools derived from several selection rounds. We observed different changes in nucleotide composition in solution PCR-driven SELEX versus ddPCR-driven SELEX (Table 11). As the selection proceeded, we noted a gradual reduction (up to 10%) in adenosine in both experiments (Fig. 4a,b). Solution PCR-driven SELEX demonstrated a progressive increase in thymidine (28.1% to 41.1%), while ddPCR-driven SELEX demonstrated a constant bias toward guanine-rich sequences throughout the entire procedure (31.2% to 34%).

Additionally, as observed previously^32^, a shift toward favorable incorporation of 2′-F pyrimidines over 2′-OH purines occurred in the solution PCR-driven SELEX (Fig. 4c). At the end of round 7, the percent of pyrimidine and purine compositions were 64% and 36%, respectively. A similar phenomenon occurred using solution PCR-driven SELEX for the CD2 target protein (data not shown). In contrast, the compositions of purine and pyrimidine were almost equal (around 50%) in the ddPCR-driven SELEX throughout the entire selection (Fig. 4d). In summary, we demonstrated that solution PCR favors the amplification of thymidine-rich sequences over others, while ddPCR preferentially incorporates guanine into the amplicon, suggesting that PCR methods account for the observed nucleotide bias.

## Discussion

HT-SELEX is currently emerging as the most fundamental advance in SELEX technology. By combining HTS with bioinformatics analysis, comprehensive sequencing data of SELEX libraries can be revealed, improving understanding of the entire selection process and facilitating rapid identification of high-affinity aptamers. Recent studies have demonstrated that HT-SELEX not only facilitates global monitoring of molecular enrichment, but also reveals inherent bias and mutations generated during the selection process^23^,^28^. In this study, we used HT-SELEX to identify several inherent biases from both chemical synthesis and PCR methods that impact the SELEX process and ultimate aptamer selection.

First we examined library complexity and nucleotide distribution in the random region of the initial, unselected RNA libraries. An increase in guanines occurred in all of the sequenced initial RNA libraries, but to a varying degree, suggesting that an inherent bias already exists in the unselected libraries. For example, deviation of nucleotide composition ranged from 0.5–4.3% in solution PCR-converted RNA libraries. This deviation was slightly higher in ddPCR-converted RNA libraries (up to 6.2%), suggesting that ddPCR causes more nucleotide biases in an initial library, with a preference toward guanosine-rich sequences. Our data also reveal that chemical synthesis of the ssDNA library is the greatest contributor to the differences in the biased sequences and their abundance. This bias in chemical synthesis affected library heterogeneity, as B-derived RNA libraries showed higher diversity than A-derived RNA libraries, while PCR method did not obviously affect library diversity. Furthermore, we believe we have shown for the first time that some biased sequences predominated in the initial, unselected library.

Next we examined the effect of biased sequences on aptamer selection. Our study demonstrates that aptamer selection and the fate of biased sequences depend on the selection target. We ultimately identified several high-affinity aptamers, suggesting that sequence bias in the initial, unselected RNA library is not so limiting as to result in the failure of aptamer selection. In the case of CD2 aptamer selection, we demonstrated that the biased sequence could be eliminated with appropriate selection stringency.

In the SELEX process, regardless of the selection of DNA or RNA aptamers, the recovered library containing bound sequences is re-amplified by PCR and subsequently converted to a new ssDNA or RNA library for the next selection cycle. It has been reported that excessive accumulation of amplification artifacts seriously hampered the enrichment of high-affinity aptamers, and even caused the failure of whole selection process^35^. However, solution PCR amplification of heterogeneous DNA libraries has revealed some limitations, such as low efficiency, formation of nonspecific product-product hybridization, and primer dimers^29^,^31^,^35^,^36^. Previous studies have demonstrated that DNA amplification by solution PCR is susceptible to nonspecific primer hybridization^29^,^31^,^37^,^38^. Musheev’s study shows that nonspecific byproducts (e.g., product-product hybridization) appear as early as the 15^th^ amplification cycle in a solution PCR^35^. By the 30^th^ cycle, all the expected products have been converted to byproducts. Although heteroduplex formation can be prevented by using excessive primers and a low number of PCR cycles, this approach is only applied to the RNA aptamer-selection. Additionally, the PCR amplification efficiency is sequence/structure-dependent. For example, it is likely more difficult to amplify highly structured DNA molecules that hinder DNA polymerase access, therefore resulting in permanent loss of these sequences. With the increase of PCR cycles and continuing selection process, the “PCR-efficient” molecules are amplified preferentially under solution PCR conditions, consequently introducing a serious bias and potential aptamer loss. In this case, “PCR-efficient” aptamer sequences, as opposed to the desired high-affinity aptamers, are overrepresented throughout the entire process.

Consistent with previous studies^28^,^30^, we showed that ddPCR-driven SELEX preserved a higher sequence diversity throughout the selection process, with molecular evolution progressing slowly (only 2-fold increase by the 5^th^ cycle). In contrast, solution PCR-driven SELEX showed a rapid convergence in library diversity (over 70-fold increase by the 5^th^ cycle), indicating a much faster enrichment rate. The overall performance of the aptamers identified from ddPCR-driven SELEX did not exceed those from solution PCR-driven SELEX. The fate of C-1A that originally existed in the initial RNA libraries is an interesting point of discussion. In both experiments, the C-1A sequence was found to be the most frequent and highly enriched aptamer, but to a varying degree. For example, by the 5^th^ selection cycle, its frequency was 85.97% in solution PCR-driven SELEX, compared to 18.81% in ddPCR-driven SELEX.

We also performed two more selection rounds after the 7^th^ round in both solution PCR driven-SELEX and ddPCR driven-SELEX experiment. The similar results were observed at the 8^th^ and 9^th^ round, in which ddPCR-driven SELEX showed lower frequencies of the top 1000 unique sequence as well as a slower molecular enrichment rate compared to solution PCR-driven SELEX (Supplementary Table S3A). For example, by the 9^th^ selection cycle, solution PCR-driven SELEX showed 775-fold molecular enrichment versus 50-fold enrichment in ddPCR-driven SELEX. Several new aptamer sequences were evolved in the additional selection cycles (Supplementary Table S3B). Two newly enriched sequences (DD9-11 and DD9-12) appeared at the 8^th^ and 9^th^ round in ddPCR-driven SELEX, while 6 newly sequences (C-2D, C-2E, C-3, C-4, C-7 and C-8) were enriched in solution PCR-driven SELEX. However, the overall binding affinity of these newly enriched sequences did not exceed the top aptamer sequences (C-2A or DD9-9) identified early by the 7^th^ round (Supplementary Figure S3B). In our study, the additional selection rounds do not benefit to aptamer selection, however they increase the cost and time of the expensive procedure. Since HT-SELEX can track the changes of aptamer family and round-to-round enrichment throughout selection cycles, it is capable of identifying candidate aptamers with high affinity at a much earlier selection round. Therefore, less number of selection cycles would be sufficient to select top aptamer candidates, which can reduce the cost and time associated with over-selection, and avoid potential PCR artifacts in the re-amplification step.

A novel finding of our study is that PCR methods make different changes in nucleotide composition as selection cycles increase. Consistent with a previous study, a gradual loss (up to 10%) in adenosine occurred in both PCR-driven SELEX experiments^32^. Compared to ddPCR-driven SELEX, which resulted in a constant bias toward guanine-rich sequences, solution PCR-driven SELEX preferentially incorporated thymidine over other nucleotides. Thiel *et al*. previously revealed that inherent bias toward pyrimidine-rich sequences appeared as early as round 2 for the both non-target and targeted selections^32^. Although they ruled out some factors that may cause nucleotide bias, such as rNTP/dNTP, SELEX target, *in vitro* transcription, and reverse transcription, the reason for the observed nucleoside bias still remained obscure, but could be due to use of the solution PCR method. Our HTS analysis of RNA libraries from ddPCR-driven SELEX sheds light on this unanswered question. We showed a similar shift toward incorporation of pyrimidines over purines in the solution PCR-driven SELEX, while purine and pyrimidine composition remained constant during ddPCR-driven selection, suggesting that PCR methods can account for the observed nucleotide bias.

Because obtaining the highest affinity and selectivity for a target is the main goal of aptamer selection, it is important to reduce bias as much as possible and maintain all possible candidates for functional testing. Otherwise, the possibility that potential aptamer candidates may remain hidden in less abundant sequences or fail to be amplified by solution PCR cannot be ruled out. For these reasons, we posit that ddPCR is a better way to preserve molecular diversity and allow more chances of obtaining highly structural sequences. However, ddPCR is costly and time-consuming compared to solution PCR. For example, ddPCR requires extra steps, including droplet generation and amplicon extraction by organic solvent from the droplets. In light of this disadvantage, an optimized solution PCR-driven SELEX may be useful. Each scientist will need to weigh the pros and cons to make an informed decision before starting a SELEX experiment. Although SELEX is a well-established process for aptamer selection, there remains room for improvement. The goal of each SELEX process is to cost-efficiently identify high-affinity aptamers for the target. Our finding clearly indicated that the initial library and PCR method are critical for the success of SELEX. We demonstrated that using HTS and bioinformatics analysis as a powerful tool to examine potential bias is an important improvement to the existing SELEX process. Our study will help researchers improve the design of future SELEX strategies and the practical success rate of effective aptamer identification.

## Methods

### PCR amplification of double-stranded (ds) DNA templates for RNA libraries

The starting ssDNA oligo library contained a 30-nt random sequence and was amplified by conventional solution PCR (S1000™ Thermal Cycler system, Bio-Rad) or ddPCR (QX200™ Droplet Digital™ PCR System, Bio-Rad) as described below. Prior to starting the selection, PCR conditions were optimized to produce the corresponding dsDNA templates (Supplementary Table S1).

#### Solution PCR

The PCR amplification protocol was 95 °C for 5 min, followed by 10 cycles of heating to 95 °C for 1 min, 63 °C for 1 min, and 72 °C 1 min. A final extension step was performed for 7 min at 72 °C. After PCR reactions (~24–48 reactions), PCR mixtures were combined and loaded on an agarose gel and the amplified dsDNA pool was recovered using a QIAquick Gel Purification Kit (Qiagen).

#### ddPCR

20 μL of each reaction mixture containing ddPCR Supermix for probes without dUTP, 3 μM forward and reverse primers, and the random sequence DNA library were loaded into the sample wells of the DG8 cartridge, followed by dispensing the droplet generator oil into each oil well in the cartridge. The cartridge was placed in the droplet generator and the resulting droplet emulsion (~40 μL) was transferred into a 96-well PCR plate. The PCR amplification protocol was as follows: 94 °C for 5 min, followed by 25 cycles of heating to 95 °C for 1 min, 63 °C for 1 min and 72 °C 1 min. A final extension step was performed for 7 min at 72 °C. After PCR reactions (~48–96 reactions), PCR mixtures were combined and excess oil was removed. PCR products were extracted from droplets using chloroform, and then loaded onto an agarose gel and recovered using a QIAquick Gel Purification Kit.

### Generation of RNA libraries and individual aptamers by T7 *in vitro* transcription

RNA libraries and individual RNA aptamers were transcribed from PCR-converted DNA templates using the DuraScribe T7 transcription Kit. In the transcription reaction mixture, the canonical cytidine triphosphate and uridine triphosphate were replaced with 2′-fluoro-cytidine triphosphate and 2′-fluoro-uridine triphosphate to produce RNA that is resistant to RNase A degradation. The reactions were incubated at 37 °C for 16 hours, and the template DNA was removed by DNase I digestion. Following phenol extraction and ethanol precipitation, the transcribed RNA libraries and RNA aptamers were purified using Bio-Spin 30 Columns. The purified RNA was quantified by ultraviolet spectrophotometry.

### Live Cell-Based SELEX

The SELEX was performed principally as described by Tuerk and Gold^2^, with application of a modified cell-based SELEX as described by Thiel *et al*.^25^. Generally, in each round, the desired amount of RNA pools were refolded in 3 mL of refolding buffer, heated to 65 °C for 5 min and then slowly cooled to 37 °C. Incubation was continued at 37 °C for 10 min. The refolding/washing buffer contained DPBS (pH 7.0 ~ 7.4), 1 mM CaCl_2_, 2.7 mM KCl, 1.47 mM KH_2_PO_4_, 1 mM MgCl_2_, 136.9 mM NaCl, 2.13 mM Na_2_HPO_4_. To avoid nonspecific interaction between nucleic acids and the cell surface, yeast tRNA (100 μg/mL in binding buffer) was used as a competitor by incubation with non-targeted cells or targeted cells at 37 °C for 25 min and then ready for selection step. A counter-selection step was performed in each cycle to minimize nonspecific binding with the non-targeted cells. Subsequently, the unbound RNA pool was transferred to the targeted cells for positive selection.

Twenty-four hours before the first cycle of selection, HeLa cells (CCR7- or CD2-negative) and HeLa-CCR7 cells (CCR7-positive) or HeLa-CD2 cells (CD2-positive) were seeded at equal density (3 × 10^6^ cells per plate) on 150 mm tissue culture dishes in 25 mL complete culture medium. On the day of selection, HeLa negative cells were washed three times with 15 mL pre-warmed washing buffer to remove dead cells and 15 mL pre-warmed binding buffer supplemented with 100 μg/mL yeast tRNAs was added. After 25 min incubation at 37 °C, the buffer was removed and the refolded RNA pool (4 nmol 0-RNA pool in 15 mL refolding buffer) was added to the HeLa negative cells for 30 min at 37 °C. This pre-cleared 0-RNA pool (supernatants with unbound sequences from negative cells plate) was then treated for positive selection. As described above, HeLa-CCR7 positive cells were also washed and incubated with 15 mL pre-warmed binding buffer supplemented with 100 μg/mL yeast tRNAs. After 25 min incubation at 37 °C, the buffer was removed and the pre-cleared 0-RNA pool was transferred to the HeLa-CCR7 positive cells for 30 min at 37 °C. Following incubation of the pre-cleared RNA pool, the HeLa-CCR7 positive cells were washed three times with 12 mL pre-warmed washing buffer to remove unbound sequences and cell-surface RNA sequences with weak binding. Cell surface-bound RNA sequences with strong binding affinity and internalized RNA sequences were recovered by TRIzol extraction (Invitrogen).

The recovered RNA pool was reverse transcribed using the ThermoScript RT-PCR system and amplified by either solution PCR or ddPCR as described above. After the amplified dsDNA template was purified using the QIAquick Gel Purification Kit, it was transcribed to the new RNA pool, as described above, for the next round of selection. As the SELEX experiments proceeded, selection conditions (e.g., cell number, density, volume, RNA amount, washing times, tRNA competitor amount, and incubation time) were adjusted to increase the pressure of aptamer selection (Supplementary Table S2).

### High throughput sequencing with Illumina HiSeq2500 and bioinformatics analysis

After 7 rounds of SELEX, the initial RNA library (0-RNA pool) and the RNA pools from selection rounds 1, 3, 5, 6 and 7 were sent for Illumina HTS analysis. Sample preparation and sequencing were performed by the City of Hope Integrative Genomics Core.

Briefly, 1.0 μg of RNA pool was first reverse-transcribed using RT primer (5′-CAG ATT GAT GGT GCC TAC AGT CGG GCG UGT CGT CTG-3′). Eight cycles of PCR amplification were performed with the primers JH5 (5′-AAT GAT ACG GCG ACC ACC GAC AGG TTC AGA GTT CGA TCG GGA GGA CGA TGC GG-3′) and RT/index primer (5′-CAG ATT GAT GGT GCC TAC AGT CGG GCG UGT CGT CTG-3′). The PCR products were purified on 6% TBE PAGE gels with size selection for targeted RNAs of 30 nt. The purified libraries were subjected to 4 cycles of second-round PCR amplification with primers PE-mi-index primer (5′-CAA GCA GAA GAC GGC ATA CGA GAT NNNNNN CAG ATT GAT GGT GCC TAC AG-3′) and R2 (5′-AAT GAT ACG GCG ACC ACC GA-3′). The final PCR products were purified by 1.8 × AMPure XP beads (Beckman Coulter) and quantified using qPCR. Library templates were prepared for sequencing using the cBot cluster generation system with HiSeq SR Cluster Kit V4 (Illumina). The sequencing run was performed in single-read mode of 51 cycles of read and 7 cycles of index read using HiSeq 2500 platform with HiSeq SBS Kit V4 (Illumina). Real-time analysis (RTA) 2.2.38 software was used to process the image analysis and base calling.

Reads processing and data analysis were conducted by the City of Hope Integrative Genomics Core facility (Dr. Xiwei Wu). In brief, the processing principles were as follows. Bases after Ns in each read were considered low quality and were removed. The 3′-fixed oligo and 3′-Solexa adapter were identified and trimmed from each read. Reads with 30-nt after processing were considered usable and retained for further analysis. Unique reads in each sample were counted and the most frequent 1,000 unique sequences were identified. The most frequent 1,000 unique sequences in round 7 were obtained and matched to the other five samples to generate the top 1,000 unique reads and their frequencies were recorded. The consensus sequence of round 7 was used to compare to the reads in each round. For alignment and grouping analysis, the top 50 sequences were divided into several groups according to their predicted secondary structures by MFold RNA (http://unafold.rna.albany.edu/?q=mfold/rna-folding-form).

### Cell-surface binding of experimental RNAs by flow cytometry analysis

Fluorescent dye-labeled RNAs were generated using the Silencer siRNA labeling kit (Ambion). For cell-surface receptor protein staining, adherent cell lines (HeLa cells, HeLa-CCR7 cells, and HeLa-CD2 cells) were washed with pre-warmed PBS and detached with Cell Stripper; suspension cell lines (Jurkat cells, H9 cells, and human memory CD4+ T cells) were washed with pre-warmed PBS. Cells were counted and the desired number of cells (2.5 × 10^5^) was resuspended in 100 μL binding buffer containing Cy3-labeled experimental RNA aptamers at different concentrations as shown in the figures. After incubation for 30 min at room temperature in the dark, cells were washed twice with 1 mL of pre-warmed binding buffer, finally resuspended in 350 μL of DPBS and processed immediately for flow cytometry (BD Fortessa, Analytical Cytometry Core, City of Hope, CA). The dissociation constants were calculated using non-liner curve regression with GraphPad Prism 6.0.

## Additional Information

**How to cite this article**: Takahashi, M. *et al*. High throughput sequencing analysis of RNA libraries reveals the influences of initial library and PCR methods on SELEX efficiency. *Sci. Rep.* **6**, 33697; doi: 10.1038/srep33697 (2016).

## Supplementary Material

Supplementary Information

## Figures and Tables

**Table 1:** Bioinformatics analysis of high throughput sequencing data from the initial RNA libraries.

A-derived initial RNA pool	A	C	T	G
Solution PCR	25.48%	23.18%	24.72%	26.63%
ddPCR	23.86%	24.67%	22.75%	28.72%
**B-derived initial RNA pool**	**A**	**C**	**T**	**G**
Solution PCR	22.50%	22.60%	28.10%	26.80%
ddPCR	19.90%	24.40%	24.50%	31.20%

ssDNA libraries purchased from different companies (A and B) were used to generate dsDNA libraries by either solution PCR or ddPCR amplification. dsDNA libraries were subsequently converted into RNA libraries via *in vitro* transcription. The nucleotide compositions (A, C, T, and G) in the 30-nt random region were identified.

**Table 2:** Bioinformatics analysis of high throughput sequencing data from the initial RNA libraries.

	A-derived Initial RNA pool		B-derived Initial RNA pool	
	Solution PCR	ddPCR	Solution PCR	ddPCR
Total Reads	28,942,595	27,863,589	40,174,712	55,173,334
Usable reads	27,411,096	26,104,315	37,951,292	51,997,175
% Usable	94.71%	93.69%	94.47%	94.24%
Total reads of top 1000 unique sequences	5,984	6,596	3,169	3,233
% top 1000 in usable reads	0.022%	0.025%	0.008%	0.006%

ssDNA libraries purchased from different companies (A and B) were used to generate dsDNA libraries by either solution PCR or ddPCR amplification. The total reads and useful reads were defined. The most frequent 1,000 unique sequences and their percent in all the usable reads were identified.

**Table 3:** Clustering analysis of RNA libraries derived from company A was used to identify related sequence groups.

Group	30-nt random sequences	A-derived initial RNA libraries					
		Reads of each groups in top 50 unique sequences		Frequency of each groups in top 1000 unique sequences		Frequency of each groups in total usable reads	
		Solution PCR	ddPCR	Solution PCR	ddPCR	Solution PCR	ddPCR
1	AATTTCCTCAACCGCGTCCATTGTCGTGTG	3,207	3,705	87.22%	88.91%	0.0117%	0.0142%
2	TCCCAACATCGTTTTAATTGTCGTAGTGTG	349	343	9.49%	8.23%	0.0013%	0.0013%
3	TTTCCCAACGCACTTCCTCGAGTTGTTGGC	87	44	2.37%	1.06%	0.0003%	0.0002%
4	AAATGTTTTTCGATCCTTTTAGTCGCTGCA	18	6	0.49%	0.14%	0.0001%	0.0000%
Others	Orphan sequences	16	69	0.44%	1.66%	0.0001%	0.0003%
	Total reads of top 50 unique sequences	3,677	4,167				

After alignment of the top 50 unique sequences, several sequence groups were identified. The representative sequences of each group, and their reads and frequencies, are listed. Only the 30-nt random sequences of the RNA core regions (5′-3′) are indicated.

**Table 4:** Clustering analysis of RNA libraries derived from company B was used to identify related sequence groups.

Group	30-nt random sequences	B-derived initial RNA libraries					
		Reads of each groups in top 50 unique sequences		Frequency of each groups in top 1000 unique sequences		Frequency of each groups in total usable reads	
		Solution PCR	ddPCR	Solution PCR	ddPCR	Solution PCR	ddPCR
1	TTTCGTCCTGAGTTCGTGTCCTCGTCTGTG	100	155	3.16%	4.79%	0.0003%	0.0003%
Others	Orphan sequences	189	186	5.96%	5.75%	0.0005%	0.0004%
	Total reads of top 50 unique sequences	289	341				

After alignment of the top 50 unique sequences, several sequence groups were identified. The representative sequences of each group, and their reads and frequencies, are listed. Only the 30-nt random sequences of the RNA core regions (5′-3′) are indicated.

**Table 5:** Bioinformatics analysis of high throughput sequencing data from selection rounds.

	Initial library	HeLa-CCR7 cell-SELEX				HeLa-CD2 cell-SELEX			
		Round 3	Round 5	Round 6	Round 7	Round 3	Round 5	Round 6	Round 7
Total Reads	40,174,712	57,383,383	49,925,077	57,778,216	51,453,049	15,346,437	16,615,193	18,644,363	21,236,560
Usable reads	37,951,292	54,654,814	45,474,572	51,503,799	44,593,424	13,682,508	14,585,023	16,629,267	19,596,020
% Usable	94.47%	95.25%	91.09%	89.14%	86.67%	89.16%	87.78%	89.19%	92.27%
Total reads of top 1000 unique sequences	3,169	5,797	226,559	564,840	771,042	326,175	6,766,517	9,237,787	16,182,954
Frequencies of top 1000 in all the usable reads	0.008%	0.011%	0.498%	1.097%	1.729%	2.384%	46.394%	55.551%	82.583%
Molecular enrichment (fold) (top 1000)	1.00	1.83	71.49	178.24	243.31	102.93	2135.22	2915.05	5106.64

The total reads and useful reads were defined. The most frequent 1,000 unique sequences and their percent in all the usable reads were identified. The molecular enrichment at each round was calculated by the formula: total reads of top 1000 unique sequences at round X/unselected round (initial library).

**Table 6:** Clustering analysis of RNA aptamers against CCR7 to identify related sequence and structure groups.

Group	CCR7 aptamer	30-nt random sequence	Reads of each group (in top 50 unique sequences)					Frequencies of each group (in top 1000 unique sequences)					
			Initial library	Round 3	Round 5	Round 6	Round 7	Initial library	Round 3	Round 5	Round 6	Round 7	
**1**	C-1A	TTTCGTCCTGAGTTCGTGTCCTCGTCTGTG	100	2,642	194,765	474,729	619,702	3.16%	45.58%	85.97%	84.05%	80.37%	↑
**2**	C-2A	AATTCGTCAAAGTCGTTTATTTCGTCTGTG	0	0	169	895	2,496	0.00%	0.00%	0.07%	0.16%	0.32%	↑
	C-2B	AATTCGTCCATTTGTCGCTCATCGTCTGTG	0	0	0	0	746	0.00%	0.00%	0.00%	0.00%	0.10%	↑
	C-2C	TTTCGTCCAATTTACGCTTCGTCGTCTGGC	0	0	0	552	1,208	0.00%	0.00%	0.00%	0.10%	0.16%	↑
**3**	C-5A	ATTTCGTCAACGCGTTTGTCTCGTCTGGTG	0	0	0	575	1,099	0.00%	0.00%	0.00%	0.10%	0.14%	↑
	C-5B	ATTCCTCAAATTCATCGATCTCGTCTGGTG	0	0	0	563	1,105	0.00%	0.00%	0.00%	0.10%	0.14%	↑
**4**	C-6	TCCATCGTCTCTTATCGTCTCTTGTCGCGC	0	24	626	1,767	2,312	0.00%	0.41%	0.28%	0.31%	0.30%	↘→
**Others**		Orphan sequences	189	84	0	0	0	5.96%	1.45%	0.00%	0.00%	0.00%	↓
		Total Reads of all the groups	289	2,750	195,560	479,081	628,668						

After alignment of the top 50 sequences, several groups of RNA aptamers were identified. The representative RNA aptamers, the reads of each group, and their frequencies in the top 1000 unique sequences were listed. Only the 30-nt random sequences of the aptamer core regions (5′-3′) are indicated.

**Table 7:** Clustering analysis of RNA aptamers against CD2 to identify related sequence and structure groups.

Group	CD2 aptamer	30-nt random sequence	Reads of each group (in top 50 unique sequences)					Frequencies of each group (in top 1000 unique sequences)					
			Initial library	Round 3	Round 5	Round 6	Round 7	Initial library	Round 3	Round 5	Round 6	Round 7	
**1**	H-1	TTTCCCAACGCACTTCCTCGAGTTGTTGGC	0	16,311	1,326,301	2,947,944	6,655,972	0.00%	5.00%	19.60%	31.91%	41.13%	↑
**2**	H-2	TCCCAACATCGTTTTAATTGTCGTAGTGTG	0	32,869	1,518,857	2,737,139	5,798,090	0.00%	10.08%	22.45%	29.63%	35.83%	↑
**3**	H-3	TCCATCGTCTCTTATCGTCTCTTGTCGCGC	0	77,956	1,202,986	1,384,237	722,365	0.00%	23.90%	17.78%	14.98%	4.46%	↓
**4**	H-4	AATTTCCTCAACCGCGTCCATTGTCGTGTG	0	15,669	763,432	148,000	770,723	0.00%	4.80%	11.28%	1.60%	4.76%	↗↘
	H-10	AATTCGTCAAAGTCGTTTATTTCGTCTGTG	0	1,118	14,494	14,547	0	0.00%	0.34%	0.21%	0.16%	0.00%	↓
**5**	H-5	AAATGTTTTTCGATCCTTTTAGTCGCTGCA	0	30,611	451,752	285,503	380,099	0.00%	9.38%	6.68%	3.09%	2.35%	↓
**6**	H-6	CACGCACGATGGATTGTTGTCTGGCCACCA	0	103,828	517,706	716,614	338,580	0.00%	31.83%	7.65%	7.76%	2.09%	↓
	H-12	TCCGCGAGTTCATTGAGTTGTTGCGCCCCA	0	1,364	0	11,418	0	0.00%	0.42%	0.00%	0.12%	0.00%	↓
**7**	H-7	ATTCCTCAAATTCATCGATCTCGTCTGGTG	0	2,580	76,991	87,753	184,665	0.00%	0.79%	1.14%	0.95%	1.14%	↓
**8**	H-8	TTTCGTCCTGAGTTCGTGTCCTCGTCTGTG	100	6,839	151,921	92,818	102,527	3.16%	2.10%	2.25%	1.00%	0.63%	↓
**9**	H-9	TCCTGGTGTTGTTGGTTGTCTGCGCTGCCC	0	5,000	25,636	38,412	38,318	0.00%	1.53%	0.38%	0.42%	0.24%	↓
**10**	H-11	TTTCGTCTTTTTATCGTTCCTATGTCGCTG	0	488	10,372	8,303	0	0.00%	0.15%	0.15%	0.09%	0.00%	↓
**11**	H-13	CACCTACACCCCGCTATTGGTCGTTGTGCA	0	317	0	0	0	0.00%	0.10%	0.00%	0.00%	0.00%	↓
**Others**		Orphan sequences	189	0	0	0	0	5.96%	0.00%	0.00%	0.00%	0.00%	↓
		Total reads of all the groups	289	294,950	6,060,448	8,472,688	14,991,339						

After alignment of the top 50 sequences, several groups of RNA aptamers were identified. The representative RNA aptamers, the reads of each group, and their frequencies in the top 1000 unique sequences were listed. Only the 30-nt random sequences of the aptamer core regions (5′-3′) are indicated.

**Table 8:** Bioinformatics analysis of high throughput sequencing data from CCR7 aptamer selections.

	Solution PCR-driven cell-SELEX						ddPCR-driven cell-SELEX					
	Initial library	Round 1	Round 3	Round 5	Round 6	Round 7	Initial library	Round 1	Round 3	Round 5	Round 6	Round 7
Total Reads	40,174,712	48,630,975	57,383,383	49,925,077	57,778,216	51,453,049	55,173,334	82,184,658	51,861,247	46,379,471	48,268,204	51,124,263
Usable reads	37,951,292	45,170,934	54,654,814	45,474,572	51,503,799	44,593,424	51,997,175	77,008,260	47,716,752	42,668,515	44,167,084	46,564,753
% Usable	94.47%	92.89%	95.25%	91.09%	89.14%	86.67%	94.24%	93.70%	92.01%	92.00%	91.50%	91.08%
Total reads of top 1000 unique sequences	3,169	3,099	5,797	226,559	564,840	771,042	3,233	3,361	3,321	7,248	15,328	35,612
Frequencies of top 1000 in all the usable reads	0.008%	0.007%	0.011%	0.498%	1.097%	1.729%	0.006%	0.004%	0.007%	0.017%	0.035%	0.076%
Molecular enrichment (fold) (top 1000)	1.00	0.98	1.83	71.49	178.24	243.31	1.00	1.04	1.03	2.24	4.74	11.02

Two cell-based selections for human CCR7 were performed in parallel using solution PCR- and ddPCR-driven HT-SELEX. The total reads and useful reads were defined as follows: The most frequent 1,000 unique sequences and their percent in all the usable reads were identified. The molecular enrichment at each round was calculated by the formula: total reads of top 1000 unique sequences at round X/unselected round (initial library).

**Table 9:** Clustering analysis of RNA aptamers in solution PCR-driven SELEX to identify related sequence and structure groups.

	Group	CCR7 aptamer	30-nt random sequence	Reads of each group(in top 50 unique sequences)						Frequencies of each group(in top 1000 unique sequences)						
				Initiallibrary	Round 1	Round 3	Round 5	Round 6	Round 7	Initiallibrary	Round 1	Round 3	Round 5	Round 6	Round 7	
Solution PCR-driven SELEX	**1**	C-1A	TTTCGTCCTGAGTTCGTGTCCTCGTCTGTG	100	96	2,642	194,765	474,729	619,702	3.16%	3.10%	45.58%	85.97%	84.05%	80.37%	↑
	**2**	C-2A	AATTCGTCAAAGTCGTTTATTTCGTCTGTG	0	0	0	169	895	2,496	0.00%	0.00%	0.00%	0.07%	0.16%	0.32%	↑
		C-2B	AATTCGTCCATTTGTCGCTCATCGTCTGTG	0	0	0	0	0	746	0.00%	0.00%	0.00%	0.00%	0.00%	0.10%	↑
		C-2C	TTTCGTCCAATTTACGCTTCGTCGTCTGGC	0	0	0	0	552	1,208	0.00%	0.00%	0.00%	0.00%	0.10%	0.16%	↑
	**3**	C-5A	ATTTCGTCAACGCGTTTGTCTCGTCTGGTG	0	0	0	0	575	1,099	0.00%	0.00%	0.00%	0.00%	0.10%	0.14%	↑
		C-5B	ATTCCTCAAATTCATCGATCTCGTCTGGTG	0	0	0	0	563	1,105	0.00%	0.00%	0.00%	0.00%	0.10%	0.14%	↑
	**4**	C-6	TCCATCGTCTCTTATCGTCTCTTGTCGCGC	0	0	24	626	1,767	2,312	0.00%	0.00%	0.41%	0.28%	0.31%	0.30%	↘→
	**Others**		Orphan sequences	189	153	84	0	0	0	5.96%	4.94%	1.45%	0.00%	0.00%	0.00%	↓
			Total Reads of all the groups	289	249	2,750	195,560	479,081	628,668							

After alignment of the top 50 sequences, several groups of RNA aptamers were identified. The representative RNA aptamers, the reads of each group, and their frequencies in the top 1000 unique sequences were listed. Only the 30-nt random sequences of the aptamer core regions (5′-3′) are indicated.

**Table 10:** Clustering analysis of RNA aptamers in ddPCR-driven SELEX to identify related sequence and structure groups.

	Group	CCR7 aptamer	30-nt random sequence	Reads of each group (top 50 unique sequences)						Frequencies of each group (in top 1000 unique sequences)						
				Initiallibrary	Round 1	Round 3	Round 5	Round 6	Round 7	Initiallibrary	Round 1	Round 3	Round 5	Round 6	Round 7	
ddPCR-drivenSELEX	**1**	C-1A	TTTCGTCCTGAGTTCGTGTCCTCGTCTGTG	155	113	191	1363	2,668	5,801	4.79%	3.36%	5.75%	18.81%	17.41%	16.29%	↑
	**2**	DD9-2	TTACTATTCAACTCGGCTCTTCGTTCTGTG	0	0	0	131	103	207	0.00%	0.00%	0.00%	1.81%	0.67%	0.58%	↑↘
	**3**	DD9-3	TCCTGGTGTTGTTGGTTGTCTGCGCTGCCC	0	0	0	0	45	147	0.00%	0.00%	0.00%	0.00%	0.29%	0.41%	↑
		DD9-7	TCCCATCGTTGAAGTTGATCTGGCCCGCCA	0	0	0	0	0	110	0.00%	0.00%	0.00%	0.00%	0.00%	0.31%	↑
		DD9-8	TCCCGAAGCATGTCGGTTGTCTGTGCGCGC	0	0	0	0	0	115	0.00%	0.00%	0.00%	0.00%	0.00%	0.32%	↑
	**4**	DD9-5	TCCCGGTGTGTAAGTTGTCTGCTGCCTGGC	0	0	0	0	40	105	0.00%	0.00%	0.00%	0.00%	0.26%	0.29%	↑
	**5**	DD9-9	TGCCGCACATTAGTCTCTGGTTGCCCACGC	0	0	0	0	0	60	0.00%	0.00%	0.00%	0.00%	0.00%	0.17%	↑
	**6**	DD9-14	TCCGCGAGTTCATTGAGTTGTTGCGCCCCA	0	0	0	0	25	62	0.00%	0.00%	0.00%	0.00%	0.16%	0.17%	↑
	**7**	C-6	TCCATCGTCTCTTATCGTCTCTTGTCGCGC	0	0	0	17	41	0	0.00%	0.00%	0.00%	0.23%	0.27%	0.00%	↘
	**Others**		Orphan sequences	186	187	192	312	828	2078	5.75%	5.56%	5.78%	4.30%	5.40%	5.84%	→
			Total Reads of all groups	341	300	383	1823	3750	8685							

After alignment of the top 50 sequences, several groups of RNA aptamers were identified. The representative RNA aptamers, the reads of each group, and their frequencies in the top 1000 unique sequences were listed. Only the 30-nt random sequences of the aptamer core regions (5′-3′) are indicated.

**Table 11:** The nucleotide compositions (A, C, T and G) in the random region were identified.

RNA library	Solution PCR-driven SELEX						ddPCR-driven SELEX					
	A	C	T	G	purine	pyrimidine	A	C	T	G	purine	pyrimidine
**Initial library**	22.50%	22.60%	28.10%	26.80%	49.30%	50.70%	19.90%	24.40%	24.50%	31.20%	51.10%	48.90%
**Round 1**	21.00%	23.30%	29.00%	26.80%	47.80%	52.30%	19.20%	24.40%	24.10%	32.20%	51.40%	48.50%
**Round 3**	18.40%	24.30%	32.90%	24.30%	42.70%	57.20%	17.90%	25.00%	25.20%	31.90%	49.80%	50.20%
**Round 5**	15.20%	24.30%	37.40%	23.10%	38.30%	61.70%	16.00%	24.50%	26.20%	33.30%	49.30%	50.70%
**Round 6**	14.40%	23.90%	39.40%	22.30%	36.70%	63.30%	15.80%	25.00%	26.20%	32.90%	48.70%	51.20%
**Round 7**	14.00%	23.00%	41.10%	22.00%	36.00%	64.10%	15.50%	24.80%	26.20%	34.00%	49.50%	51.00%

The initial RNA libraries and several RNA libraries from selection rounds 1, 3, 5, 6 and 7 in solution PCR-driven SELEX or ddPCR-driven SELEX were analyzed by high throughput sequencing.
