# Supplementary material for: High throughput sequencing analysis of RNA libraries reveals the influences of initial library and PCR methods on SELEX efficiency
Source: Sci Rep. 2016 Sep 22;6:33697. doi: 10.1038/srep33697 (PMC5031971; doi:10.1038/srep33697)
Supplement: Supplementary Information [file srep33697-s1.pdf]

# High throughput sequencing analysis of RNA libraries reveals the influences of initial library and PCR methods on SELEX efficiency

Mayumi Takahashi[1], Xiwei Wu[1, 2], Michelle Ho[3], Pritsana Chomchan[1],, John J. Rossi[1, 3], John C. Burnett[1*] and Jiehua Zhou[1*]

[1] Department of Molecular and Cellular Biology, Beckman Research Institute of City of Hope, Duarte, CA, 91010, USA

[2] Integrative Genomics Core, Beckman Research Institute of City of Hope, Duarte, CA, 91010, USA

[3] Irell and Manella Graduate School of Biological Sciences, Beckman Research Institute of City of Hope, Duarte, CA, 91010, USA

*Correspondence should be addressed to John Burnett. Tel: (1)-6263019035; Fax: (1)-6263018862; Email: jburnett@coh.org; Correspondence also be addressed to Jiehua Zhou. Tel: (1)-6262180533; Fax: (1)-6263018271; Email: jzhou@coh.org;

# Supplementary information

## MATERIALS AND METHODS:

### Materials

Unless otherwise noted, all chemicals were purchased from Sigma-Aldrich, all restriction enzymes were obtained from New England BioLabs, and all cell culture products were purchased from Meditech (Meditech, a division of CORNING). Sources for the other reagents were: Taq DNA polymerase (Sigma); ddPCR supermixes and droplet generation oil (Bio-Rad); DuraScribe T7 transcription Kit (EPICENTRE Biotechnologies); ThermoScript RT-PCR system (Invitrogen); Silencer siRNA Labeling Kit (Ambion); Hoechst 33342 (nuclear dye for live cells; Molecular Probes, Invitrogen); M-MLV reverse transcriptase and random primers (Invitrogen); Bio-Spin 30 Columns (Bio-Rad); Lipofectamine 2000 (Invitrogen); Recombinant CCR7 protein (ORIGENE).

Initial 61-nucleotide (nt) ssDNA oligo libraries with 30-nt random sequences were purchased from two different manufacturers. The sequence of the 61-mer ssDNA oligo libraries is: 5'- GGG AGG ACG ATG CGG – $N_{30}$ – CAG ACG ACT CGC CCG A -3' (61 nt). The random region is flanked by constant regions. Primers were purchased from Integrated DNA Technologies (IDT). Forward primer: 5'- TAA TAC GAC TCA CTA TAG GGA GGA CGA TGC GG -3' (32 mer, T7 promoter is underlined); Reverse primer: 5'- TCG GGC GAG TCG TCT G -3' (16 mer).

### Generation of cell line stably expressing the selection targets

HeLa-CCR7 and HeLa-CD2 cells were generated by lentivirus vector-mediated delivery of CCR7 or CD2 genes, respectively. The CCR7 (pFUG-P2A-CCR7-W) or CD2 (pFUG-P2A-CD2-W) gene expression plasmids were generated by digesting the pFUGW lentiviral plasmid (a gift from David Baltimore) with BsrGI-EcoRI restriction sites and inserting custom-designed P2A-CCR7 or P2A-CD2 gBlock gene fragments (IDT) into same sites. The lentiviral plasmids were packaged and harvested in HEK293T cells using 10 µg of vector, 5 µg pMDLg/pRRE, 3.5 µg pVSV-G, and 1.5 µg pRSV-Rev. Viral supernatant was collected 24 hours after transfection and passed through a 0.45-µm filter to remove cell debris. The virus was then loaded onto a 20% (wt/wt) sucrose cushion and concentrated by ultracentrifugation in an SW28 rotor on Optima XL-100K Ultracentrifuge (Beckman Coulter) for 1.5 hours at 112,000 *g* at 4 ° C. The viral pellet was resuspended in 100 µl of phosphate-buffered saline (pH 7.0). The resulting lentivirus was transduced into HeLa cells. Three weeks after transduction, GFP-positive cells were sorted with fluorescence activated cell sorting.

### Cell lines and cell culture

All cells were cultured in a humidified 5% $CO_2$ incubator at 37° C.

*HeLa cell lines* (ATCC). Adherent HeLa cell lines were cultured in 90% DMEM supplemented with 10% fetal bovine serum and 1% glutamine. Cells were split 1:10 or 1:5 once per week upon reaching confluence by washing with DPBS and detaching cells using Cell Stripper (Cellgro, Mediatech Inc) in order to minimize the damage to the cellular surface receptors. Cells were stained with Trypan Blue to quantitate viability. Cells with >95% viability were used for seeding. Each cell line was carried for no more than 20 passages.

*Jurkat and H9 cells* (ATCC). Jurkat and H9 suspension cell lines were cultured in RPMI-1640 supplemented with 10% fetal bovine serum. Cells were split 1:10 once per week.

*Human memory CD4+ T cells*. Peripheral blood mononuclear cells (PBMCs) were obtained from healthy donors at City of Hope National Medical Center using discarded anonymous blood unit leukocyte filters (Pall). Human $CD4^+$ $T_{CM}$ cells were isolated from PBMCs using CD62L positive selection, followed by $CD4^+$ memory T cells isolation (StemCell Technologies, BC Canada). The cells were maintained in RPMI-1640 with 60 units/mL IL-2. The cell surface markers on the isolated cells were characterized with various antibodies by flow cytometry.

We use discarded peripheral blood from anonymous adult donors from the City of Hope Apheresis Center (Duarte, CA) for primary $CD4^+$ T cell cultures. The proposed research involves blood specimens from anonymous human subjects with no identifiers to age, race, ethnicity, or gender.

The information provided for the above submission was evaluated and determined to not involve human subjects research (45 CFR 46.102 (d)(f)). Therefore, it does not need to be approved nor does it need to undergo continuing review by the Institutional Review Board (IRB) in the City of Hope. IRB#/REF#: 97071 / 075546.

## MATERIALS AND METHODS:

### Detection of CCR7 and CD2 cell surface target protein expression by flow cytometry analysis

For cell-surface receptor staining, adherent cell lines (HeLa cells, HeLa-CCR7 cells, and HeLa-CD2 cells) were washed with pre-warmed PBS and detached with Cell Stripper. Suspension cell lines (Jurkat cells, H9 cells, and human memory CD4+ T cells) were washed with pre-warmed PBS. Cells were counted and the desired numbers of cells (e.g. $2.5\times10^5$) were resuspended in 100 μL PBS binding buffer with added PE-CF594-conjugated anti-human CCR7 antibody or PE conjugated anti-human CD2 antibody (BD Biosciences). 1 μL antibody was used per $2\times10^5$ cells in a 100 μL reaction system. After incubation for 30 min at room temperature in the dark, cells were washed twice with 1 mL of washing buffer, resuspended in 350 μL of DPBS, and processed immediately for flow cytometry (BD Fortessa, Flow Cytometry Core, City of Hope, CA).

**Gel shift assay.** The recombinant CCR7 protein was serially diluted to the desired concentrations (0 ~ 3200 nM). A constant amount of $P^{32}$ end-labeled RNA (2 nM) was used. The samples were incubated for 30 min at room temperature. After incubation, 20 μL of binding reaction was loaded into a 5% non-denaturing polyacrylamide gel. Following electrophoresis at cold room, the gel was exposed to a Phosphorimage screen and the radioactivity was quantified using a Typhoon scanner.

### Statistical analysis

GraphPad Prism 6.0 software was used for data analysis (Student's t-test). Unless otherwise noted, when error bars are indicated within figures, they represent standard deviation (SD). One-site binding (hyperbola) is used for the calculation of $K_d$ value, that is the concentration of ligand required to reach half-maximal binding. One-site binding describes the binding of a ligand to a receptor that follows the law of mass action.

## TABLE AND FIGURE LEGENDS:

**Table S1: The optimized PCR amplification of double-stranded DNA templates for RNA libraries.** The starting single-stranded DNA oligo library contained 30 nt of random sequences and was amplified by conventional solution PCR (S1000™ Thermal Cycler system, Bio-Rad) and droplet PCR (QX200™ Droplet Digital™ PCR System, Bio-Rad), respectively. Prior to starting the selection, PCR condition was optimized to get the corresponding double-stranded DNA templates.

**Table S2: The selection condition used in CCR7 or CD2 aptamers selection.** The numbers of cells, plate size, medium volume, the amount of RNA pool and tRNA, washing condition, and incubation time are indicated here.

**Table S3: Bioinformatics analysis of high throughput sequencing data from CCR7 aptamer selection.** Two cell-based selections for human CCR7 were performed in parallel using solution PCR- and ddPCR-driven HT-SELEX. (A) The total reads and useful reads were defined as follows: The most frequent 1,000 unique sequences and their percent in all the usable reads were identified. The molecular enrichment at each round was calculated by the formula: total reads of top 1000 unique sequences at round X / unselected round (initial library). (B) Clustering analysis of RNA aptamers in solution PCR-driven SELEX or ddPCR-driven SELEX to identify related sequence and structure groups. After alignment of the top 50 sequences, several groups of RNA aptamers were identified. The representative RNA aptamers, the reads of each group, and their frequencies in the top 1000 unique sequences were listed. Only the 30-nt random sequences of the aptamer core regions (5'-3') are indicated.

**Figure S1: Nucleotide distribution in the 30-nt random region of the initial RNA libraries.** Solution PCR or ddPCR was used to generate dsDNA template that was subsequently converted to RNA libraries for HTS analysis.

**Figure S2: Nucleotide distribution in the 30-nt random region of each selection rounds.** (A) HeLa-CCR7 cell-SELEX and (B) HeLa-CD2 cell-SELEX.

**Figure S3:** (A) Distribution of frequencies of top 1000 unique sequences at each round. The most frequent 1,000 unique sequences were identified at each selection round in HeLa-CCR7 HT-SELEX. From Round 5, a significant enrichment was observed in both solution PCR –driven and ddPCR-driven selections. (B) The selected CCR7 aptamers specifically bind to CCR7 protein and CCR7 expressing cells. Cell surface binding of Cy3-labeled RNAs was assessed by flow cytometry and the protein binding affinity was tested by gel shift assay.

# Table S1

| PCR condition | Solution PCR | Droplet Digital PCR |
|---|---|---|
| **Instrument** | S1000™ Thermal Cycler system (Bio-Rad) | QX200™ Droplet Digital™ PCR System (Bio-Rad) |
| **Reaction volume** | 50 µL per reaction | 20 µL per reaction (40 µL total w/oil) |
| **Primers** | 3 µM | 3 µM |
| **DNA oligo template** | 10 pmol | 5 pmol |
| **DNA polymerase / other components** | 0.4 µL (Sigma Taq); 2 mM $MgCl_2$; 200 µM of each dNTP; | ddPCR supermix (Bio-Rad) |
| **# of PCR cycles** | 10-15 cycles | 25 cycles |
| **PCR program** | 94°C, 5 min;<br>94°C, 1 min; 63°C, 1 min; 72°C, 1 min;<br>72°C, 7 min; | 94°C, 5 min;<br>94°C, 1 min; 63°C, 1 min; 72°C, 1 min;<br>72°C, 7 min; |

# Table S2

| SELEX rounds | Positive cells (plate size and medium volume) | Negative cells (plate size and medium volume) | RNA pool (incubation time) | RNA work Con. (nM) | Competitor tRNA (nmol) | Washing |
|---|---|---|---|---|---|---|
| **1** | $3*10^6$ cells (15 cm, 15 mL) | $3*10^6$ cells (15 cm, 15 mL) | 4 nmol (30 min) | 267 | 0 | 3 × 12 mL |
| **2** | $3*10^6$ cells (15 cm, 15 mL) | $3*10^6$ cells (15 cm, 15 mL) | 3 nmol (25 min) | 200 | 2.5 | 4 × 12 mL |
| **3** | $1.5*10^6$ cells (10 cm, 12 mL) | $3*10^6$ cells (10 cm, 12 mL) | 2 nmol (25 min) | 167 | 5 | 5 × 12 mL |
| **4** | $1.5*10^6$ cells (10 cm, 12 mL) | $3*10^6$ cells (10 cm, 12 mL) | 2 nmol (20 min) | 167 | 15 | 6 × 12 mL |
| **5** | $6*10^5$ cells (6 cm, 8 mL) | $2.25*10^6$ cells (10 cm, 8 mL) | 1 nmol (20 min) | 125 | 15 | 8 × 10 mL |
| **6** | $6*10^5$ cells (6 cm, 8 mL) | $2.25*10^6$ cells (10 cm, 8 mL) | 1 nmol (15 min) | 125 | 20 | 9 × 10 mL |
| **7** | $2.5*10^5$ cells (3.5 cm, 5 mL) | $2.25*10^6$ cells (10 cm, 5 mL) | 0.5 nmol (15 min) | 100 | 20 | 10 × 5 mL |

# Table S3A

| CCR7 aptamer | Solution PCR-driven cell-SELEX | | | | | | | |
|---|---|---|---|---|---|---|---|---|
| | Initial library | Round 1 | Round 3 | Round 5 | Round 6 | Round 7 | Round 8 | Round 9 |
| Total Reads | 40,174,712 | 48,630,975 | 57,383,383 | 49,925,077 | 57,778,216 | 51,453,049 | 53,345,565 | 47,815,903 |
| Usable reads | 37,951,292 | 45,170,934 | 54,654,814 | 45,474,572 | 51,503,799 | 44,593,424 | 43,801,692 | 38,523,003 |
| % Usable | 94.47% | 92.89% | 95.25% | 91.09% | 89.14% | 86.67% | 82.11% | 80.57% |
| | | | | | | | | |
| Total reads of top 1000 unique sequences | 3,169 | 3,099 | 5,797 | 226,559 | 564,840 | 771,042 | 1,566,586 | 2,457,378 |
| Frequencies of top 1000 in all the usable reads | 0.008% | 0.007% | 0.011% | 0.498% | 1.097% | 1.729% | 3.577% | 6.379% |
| Molecular enrichment (fold) (top 1000) | 1.00 | 0.98 | 1.83 | 71.49 | 178.24 | 243.31 | 494.35 | 775.44 |
| **CCR7 aptamer** | **ddPCR-driven cell-SELEX** | | | | | | | |
| | Initial library | Round 1 | Round 3 | Round 5 | Round 6 | Round 7 | Round 8 | Round 9 |
| Total Reads | 55,173,334 | 82,184,658 | 51,861,247 | 46,379,471 | 48,268,204 | 51,124,263 | 53,382,127 | 51,818,881 |
| Usable reads | 51,997,175 | 77,008,260 | 47,716,752 | 42,668,515 | 44,167,084 | 46,564,753 | 48,654,480 | 46,985,618 |
| % Usable | 94.24% | 93.70% | 92.01% | 92.00% | 91.50% | 91.08% | 91.14% | 90.67% |
| | | | | | | | | |
| Total reads of top 1000 unique sequences | 3,233 | 3,361 | 3,321 | 7,248 | 15,328 | 35,612 | 79,845 | 161,871 |
| Frequencies of top 1000 in all the usable reads | 0.006% | 0.004% | 0.007% | 0.017% | 0.035% | 0.076% | 0.164% | 0.345% |
| Molecular enrichment (fold) (top 1000) | 1.00 | 1.04 | 1.03 | 2.24 | 4.74 | 11.02 | 24.70 | 50.07 |

# Table S3B

| | Groups | CCR7 aptamer | 30-nt random sequence | Reads of each group (in top 50 unique sequences) | | | | | | | | Frequencies of each group (in top 1000 unique sequences) | | | | | | | | |
|---|---|---|---|---|---|---|---|---|---|---|---|---|---|---|---|---|---|---|---|---|
| | | | | Initial library | Round 1 | Round 3 | Round 5 | Round 6 | Round 7 | Round 8 | Round 9 | Initial library | Round 1 | Round 3 | Round 5 | Round 6 | Round7 | Round 8 | Round 9 | |
| Solution PCR-driven SELEX | 1 | C-1A | TTTCGTCCTGAGTTCGTGTCCTCGTCTGTG | 100 | 96 | 2,642 | 194,765 | 474,729 | 619,702 | 1,235,076 | 1,917,861 | 3.16% | 3.10% | 45.58% | 85.97% | 84.05% | 80.37% | 78.84% | 78.05% | ↑ |
| | 2 | C-2A | AATTCGTCAAAGTCGTTTATTTCGTCTGTG | 0 | 0 | 0 | 169 | 895 | 2,496 | 7,154 | 15,075 | 0.00% | 0.00% | 0.00% | 0.07% | 0.16% | 0.32% | 0.46% | 0.61% | ↑ |
| | | C-2B | AATTCGTCCATTTGTCGCTCATCGTCTGTG | 0 | 0 | 0 | 0 | 0 | 746 | 1354 | 2450 | 0.00% | 0.00% | 0.00% | 0.00% | 0.00% | 0.10% | 0.09% | 0.10% | ↑ |
| | | C-2C | TTTCGTCCAATTTACGCTTCGTCGTCTGGC | 0 | 0 | 0 | 0 | 552 | 1,208 | 3,315 | 6,256 | 0.00% | 0.00% | 0.00% | 0.00% | 0.10% | 0.16% | 0.21% | 0.25% | ↑ |
| | | C-2D | AATTTCCTCAACCGCGTCCATTGTCGTGTG | 0 | 0 | 0 | 0 | 0 | 0 | 2,443 | 4,561 | 0.00% | 0.00% | 0.00% | 0.00% | 0.00% | 0.00% | 0.16% | 0.19% | ↑ |
| | | C-2E | AATTTCCGTCCTGAATTGTTGTTGTCTGTG | 0 | 0 | 0 | 0 | 0 | 0 | 0 | 2,266 | 0.00% | 0.00% | 0.00% | 0.00% | 0.00% | 0.00% | 0.00% | 0.09% | ↑ |
| | 3 | C-5A | ATTTCGTCAACGCGTTTGTCTCGTCTGGTG | 0 | 0 | 0 | 0 | 575 | 1,099 | 0 | 3,242 | 0.00% | 0.00% | 0.00% | 0.00% | 0.10% | 0.14% | 0.00% | 0.13% | ↑ |
| | | C-5B | ATTCCTCAAATTCATCGATCTCGTCTGGTG | 0 | 0 | 0 | 0 | 563 | 1,105 | 2,394 | 3,196 | 0.00% | 0.00% | 0.00% | 0.00% | 0.10% | 0.14% | 0.15% | 0.13% | ↑ |
| | 4 | C-6 | TCCATCGTCTCTTATCGTCTCTTGTCGCGC | 0 | 0 | 24 | 626 | 1,767 | 2,312 | 3,447 | 2,661 | 0.00% | 0.00% | 0.41% | 0.28% | 0.31% | 0.30% | 0.22% | 0.11% | ↘→ |
| | 5 | C-3 | TTTCGTCTTTTTATCGTTCCTATGTCGCTG | 0 | 0 | 0 | 0 | 0 | 0 | 1,579 | 2,805 | 0.00% | 0.00% | 0.00% | 0.00% | 0.00% | 0.00% | 0.10% | 0.11% | ↑ |
| | 6 | C-4 | TTTCCCAACGCACTTCCTCGAGTTGTTGGC | 0 | 0 | 0 | 0 | 0 | 0 | 0 | 3,207 | 0.00% | 0.00% | 0.00% | 0.00% | 0.00% | 0.00% | 0.00% | 0.13% | ↑ |
| | 7 | C-7 | AAATGTTTTTCGATCCTTTTAGTCGCTGCA | 0 | 0 | 0 | 0 | 0 | 0 | 0 | 2,414 | 0.00% | 0.00% | 0.00% | 0.00% | 0.00% | 0.00% | 0.00% | 0.10% | ↑ |
| | 8 | C-8 | TCCCAACATCGTTTTAATTGTCGTAGTGTG | 0 | 0 | 0 | 0 | 0 | 0 | 0 | 2,234 | 0.00% | 0.00% | 0.00% | 0.00% | 0.00% | 0.00% | 0.00% | 0.09% | ↑ |
| | Others | | Orphan sequences | 189 | 153 | 84 | 0 | 0 | 0 | 0 | 0 | 5.96% | 4.94% | 1.45% | 0.00% | 0.00% | 0.00% | 0.00% | 0.00% | |
| | | | Total Reads of all the groups | 289 | 249 | 2,750 | 195,560 | 479,081 | 628,668 | 1,256,762 | 1,968,228 | | | | | | | | | |

| | Groups | CCR7 aptamer | 30-nt random sequence | Reads of each group (top 50 unique sequences) | | | | | | | | Frequencies of each group (in top 1000 unique sequences) | | | | | | | | |
|---|---|---|---|---|---|---|---|---|---|---|---|---|---|---|---|---|---|---|---|---|
| | | | | Initial library | Round 1 | Round 3 | Round 5 | Round 6 | Round 7 | Round 8 | Round 9 | Initial library | Round 1 | Round 3 | Round 5 | Round 6 | Round 7 | Round 8 | Round 9 | |
| ddPCR-driven SELEX | 1 | C-1A | TTTCGTCCTGAGTTCGTGTCCTCGTCTGTG | 155 | 113 | 191 | 1363 | 2,668 | 5,801 | 9,403 | 14,160 | 4.79% | 3.36% | 5.75% | 18.81% | 17.41% | 16.29% | 11.78% | 8.75% | ↑↘ |
| | 2 | DD9-2 | TTACTATTCAACTCGGCTCTTCGTTCTGTG | 0 | 0 | 0 | 131 | 103 | 207 | 0 | 2035 | 0.00% | 0.00% | 0.00% | 1.81% | 0.67% | 0.58% | 0.00% | 1.26% | ↑↘ |
| | 3 | DD9-3 | TCCTGGTGTTGTTGGTTGTCTGCGCTGCCC | 0 | 0 | 0 | 0 | 45 | 147 | 445 | 1182 | 0.00% | 0.00% | 0.00% | 0.00% | 0.29% | 0.41% | 0.56% | 0.73% | ↑ |
| | | DD9-7 | TCCCATCGTTGAAGTTGATCTGGCCCGCCA | 0 | 0 | 0 | 0 | 0 | 110 | 287 | 860 | 0.00% | 0.00% | 0.00% | 0.00% | 0.00% | 0.31% | 0.36% | 0.53% | ↑ |
| | | DD9-8 | TCCCGAAGCATGTCGGTTGTCTGTGCGCGC | 0 | 0 | 0 | 0 | 0 | 115 | 306 | 726 | 0.00% | 0.00% | 0.00% | 0.00% | 0.00% | 0.32% | 0.38% | 0.45% | ↑ |
| | 4 | DD9-5 | TCCCGGTGTGTAAGTTGTCTGCTGCCTGGC | 0 | 0 | 0 | 0 | 40 | 105 | 482 | 988 | 0.00% | 0.00% | 0.00% | 0.00% | 0.26% | 0.29% | 0.60% | 0.61% | ↑ |
| | 5 | DD9-9 | TGCCGCACATTAGTCTCTGGTTGCCCACGC | 0 | 0 | 0 | 0 | 0 | 60 | 220 | 629 | 0.00% | 0.00% | 0.00% | 0.00% | 0.00% | 0.17% | 0.28% | 0.39% | ↑ |
| | 6 | DD9-14 | TCCGCGAGTTCATTGAGTTGTTGCGCCCCA | 0 | 0 | 0 | 0 | 25 | 62 | 185 | 517 | 0.00% | 0.00% | 0.00% | 0.00% | 0.16% | 0.17% | 0.23% | 0.32% | ↑ |
| | 7 | C-6 | TCCATCGTCTCTTATCGTCTCTTGTCGCGC | 0 | 0 | 0 | 17 | 41 | 0 | 0 | 0 | 0.00% | 0.00% | 0.00% | 0.23% | 0.27% | 0.00% | 0.00% | 0.00% | ↓ |
| | 8 | DD9-11 | CACCTACACCCCGCTATTGGTCGTTGTGCA | 0 | 0 | 0 | 0 | 0 | 0 | 226 | 541 | 0.00% | 0.00% | 0.00% | 0.00% | 0.00% | 0.00% | 0.28% | 0.33% | ↑ |
| | 9 | DD9-12 | CACGCACGATGGATTGTTGTCTGGCCACCA | 0 | 0 | 0 | 0 | 0 | 0 | 0 | 519 | 0.00% | 0.00% | 0.00% | 0.00% | 0.00% | 0.00% | 0.00% | 0.32% | ↑ |
| | Others | | Orphan sequences | 186 | 187 | 192 | 312 | 828 | 2078 | 5,868 | 13,081 | 5.75% | 5.56% | 5.78% | 4.30% | 5.40% | 5.84% | 7.35% | 8.08% | → |
| | | | Total Reads of all groups | 341 | 300 | 383 | 1823 | 3750 | 8,685 | 17,422 | 35,238 | | | | | | | | | |

# Figure S1

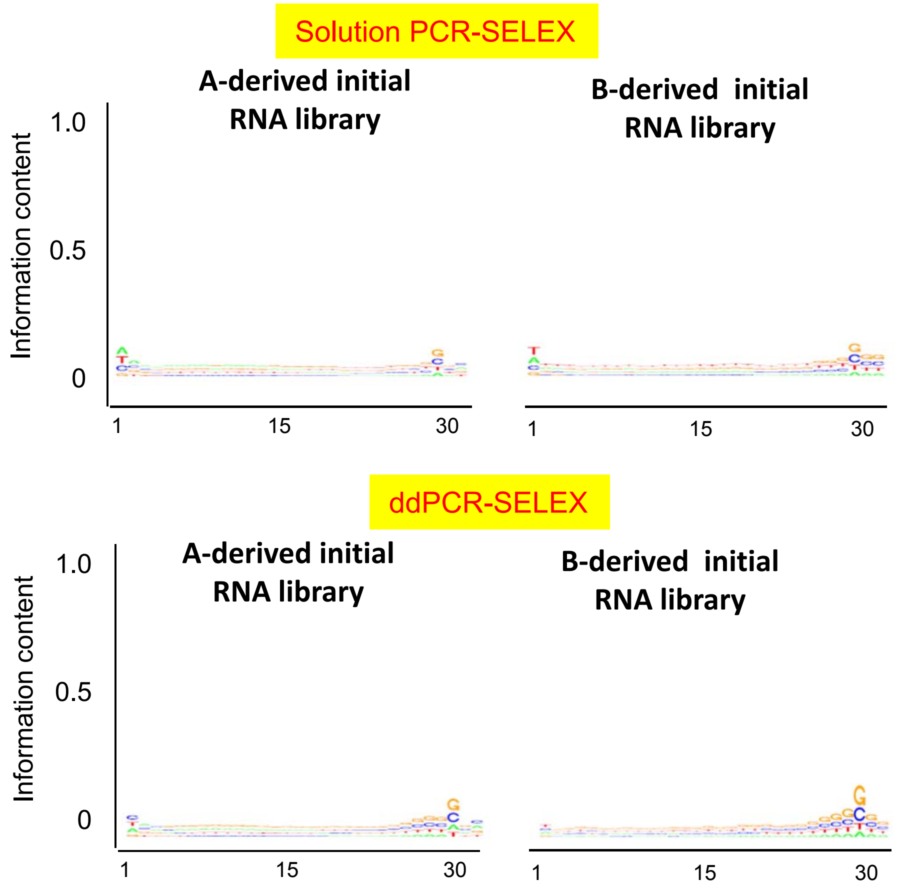
Solution PCR-SELEX
A-derived initial RNA library
B-derived initial RNA library
Information content
1.0
0.5
0
1
15
30
ddPCR-SELEX
A-derived initial RNA library
B-derived initial RNA library
Information content
1.0
0.5
0
1
15
30

# Figure S2A

Solution PCR-driven Cell-SELEX

Distribution of nucleotides in random sequence (HeLa-CCR7 cell-SELEX)

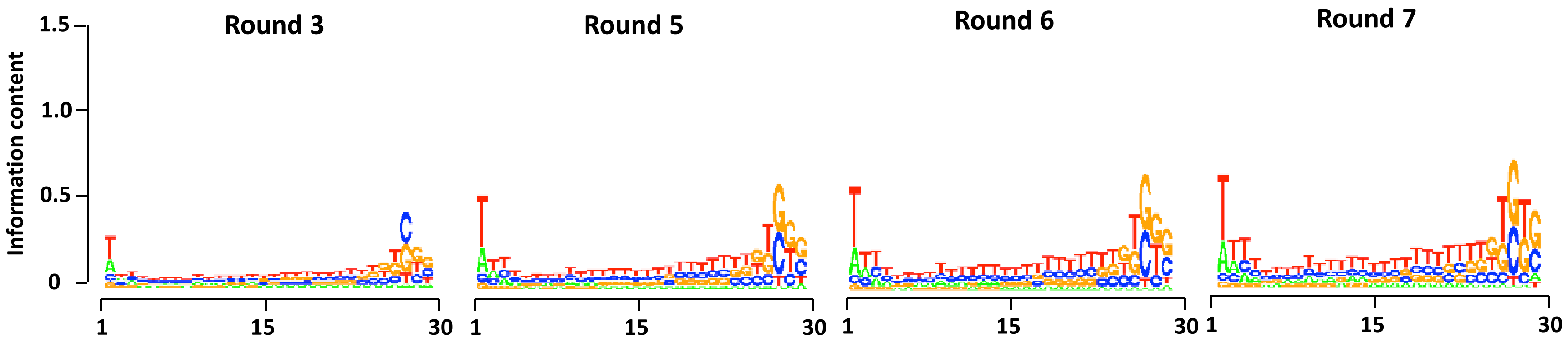


# Figure S2B

Distribution of nucleotides in random sequence (HeLa-CD2 cell-SELEX)

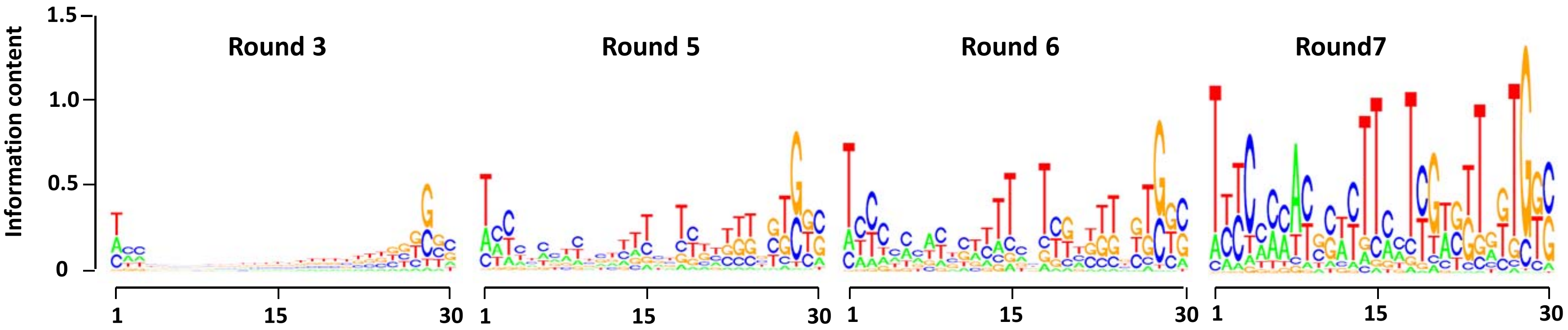

# Figure S3A

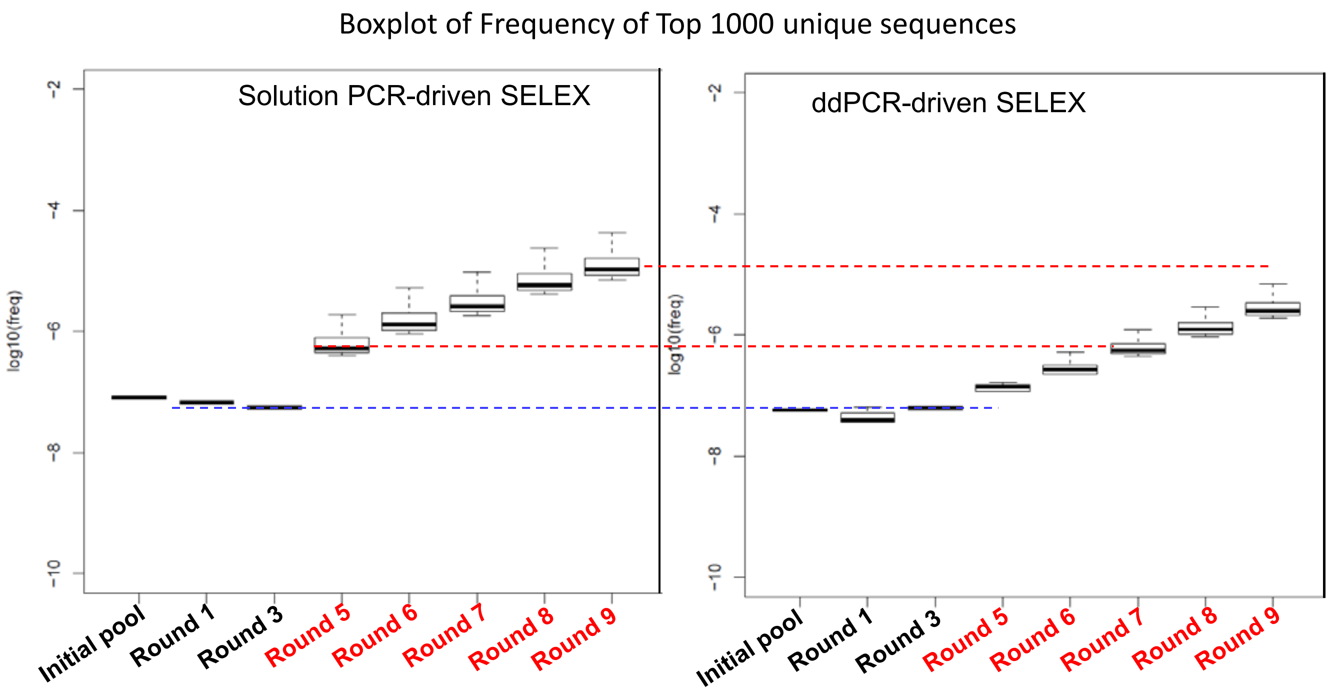

Boxplot of Frequency of Top 1000 unique sequences
Solution PCR-driven SELEX
ddPCR-driven SELEX
log10(freq)
-2
-4
-6
-8
-10
Initial pool
Round 1
Round 3
Round 5
Round 6
Round 7
Round 8
Round 9

# Figure S3B

| SELEX | CCR7 aptamers | Flow cytometry analysis (CCR7 positive cells) | | | Gel shift assay |
|---|---|---|---|---|---|
| | | HeLa-CCR7 cells | H9 cells | Human memory CD4+ T cells | Recombinant CCR7 protein |
| Solution PCR-driven SELEX | C-1A | +++ | +++ | ++ | ++ |
| | C-2A | +++ | +++ | ++ | +++ |
| | C-5B | + | +++ | + | + |
| | C-6 | ++ | ++ | + | + |
| | C-2D | ++ | ++ | + | + |
| | C-3 | ++ | +++ | + | + |
| | C-4 | ++ | ++ | + | +++ |
| | C-7 | + | + | + | ++ |
| | C-8 | ++ | ++ | ++ | |
| ddPCR-driven SELEX | DD9-2 | + | ++ | ++ | + |
| | DD9-3 | + | + | ++ | + |
| | DD9-5 | ++ | ++ | ++ | + |
| | DD9-9 | +++ | ++ | ++ | ++ |
| | DD9-14 | + | + | + | ++ |
| | DD9-11 | + | ++ | ++ | + |
| | DD9-12 | + | + | + | + |

**+++: >50% binding affinity**
**++: 30-50% binding affinity**
**+: 10-25% binding affinity**
